# Predicting the level of anemia among Ethiopian pregnant women using homogeneous ensemble machine learning algorithm

**DOI:** 10.1186/s12911-022-01992-6

**Published:** 2022-09-22

**Authors:** Belayneh Endalamaw Dejene, Tesfamariam M. Abuhay, Dawit Shibabaw Bogale

**Affiliations:** College of Informatics, University of Gondar, Gondar, Ethiopia

**Keywords:** Homogeneous ensemble machine learning, Health informatics, Anemia, Maternal healthcare

## Abstract

**Background:**

More than 115,000 maternal deaths and 591,000 prenatal deaths occurred in the world per year with anemia, the reduction of red blood cells or hemoglobin in the blood. The world health organization divides anemia in pregnancy into mild anemia (Hb 10–10.9 g/dl), moderate anemia (Hb 7.0–9.9 g/dl), and severe anemia (Hb < 7 g/dl). This study aims to predict the level of anemia among pregnant women in the case of Ethiopia using homogeneous ensemble machine learning algorithms.

**Methods:**

This study was conducted following a design science approach. The data were gathered from the Ethiopian demographic health survey and preprocessed to get quality data that are suitable for the machine learning algorithm to develop a model that predicts the levels of anemia among pregnant. Decision tree, random forest, cat boost, and extreme gradient boosting with class decomposition (one versus one and one versus rest) and without class decomposition were employed to build the predictive model. For constructing the proposed model, twelve experiments were conducted with a total of 29,104 instances with 23 features, and a training and testing dataset split ratio of 80/20.

**Results:**

The overall accuracy of random forest, extreme gradient boosting, and cat boost without class decompositions is 91.34%, 94.26%, and 97.08.90%, respectively. The overall accuracy of random forest, extreme gradient boosting, and cat boost with one versus one is 94.4%, 95.21%, and 97.44%, respectively. The overall accuracy of random forest, extreme gradient boosting, and cat boost with one versus the rest are 94.4%, 94.54%, and 97.6%, respectively.

**Conclusion:**

Finally, the researcher decided to use cat boost algorithms with one versus the rest for further use in the development of artifacts, model deployment, risk factor analysis, and generating rules because it has registered better performance with 97.6% accuracy. The most determinant risk factors of anemia among pregnant women were identified using feature importance. Some of them are the duration of the current pregnancy, age, source of drinking water, respondent’s (pregnant women) occupation, number of household members, wealth index, husband/partner's education level, and birth history.

## Background

According to [1], Anemia is defined as a decrease in the number of red blood cell or hemoglobin in the blood that has significant adverse health consequences. Anemia is a public health problem among women of reproductive age, affecting both poor and rich countries overall the world [2]. It negatively affects the social and economic well-being of a country and its communities [1]. According to [3] and [4], anemia during pregnancy is one of the risk factors for poor pregnancy outcomes such as low birth weight (LBW), preterm birth, prematurity stillbirth, intrauterine growth restriction, and impaired cognitive development.

Anemia in pregnant women can be caused by parasitic infestation, socio-demographic status, economic status, dietary practice, obstetric factors, reproductive health, and other health-related factors [5]. More than 115,000 maternal deaths and 591,000 prenatal deaths are caused by anemia disease in the world per year [6]. According to the World Health Organization (WHO, 1993–2005) report, anemia affects 41.8% of pregnant women worldwide, with Africa having the highest prevalence (57.1%) [7, 8]. According to [4] and [9], anemia during pregnancy is the main cause of morbidity and mortality of pregnant women in developing countries like Ethiopia and has both maternal and fetal consequences such as impairment of the capacity of the blood to transport oxygen around the body, fatigue, poor work capacity, impaired immune function, increased risk of cardiac diseases, and mortality [4, 10]. The burden and underlying factors of this disease varied even within a country [10]. Most of the women who live in the rural areas of Ethiopia have been affected by this disease due to different factors including nutrition, parasites, socio-demographic, obstetric, reproductive characteristics, and the like [10]. According to WHO guidelines, the minimum acceptable hemoglobin level during pregnancy is 11 g/dl, during the first half, 10.5 g/dl, during the second half, and 12 g/dl for lactating women [6, 10, 11]. To understand and predict the level of anemia among pregnant women in the case of Ethiopia, several types of research have been conducted. For example, [3, 6–14] investigated the status of anemia among pregnant women using cross-sectional statistical methods. They also used bivariate and multivariate logistic regression methods and identified the most determinant risk factors. Most of these studies, however, used local clinical data that covered limited geographical areas like a single city or town only, small data set of less than 500 instances/records, and only focused on one of the risk factors such as socioeconomic, demographic, nutritional, and reproductive, apart from health-related variables. Previous studies including [3, 6–14] also focused on identifying the determinant risk factors of anemia among pregnant women who followed first antenatal care only using descriptive statistical models. Besides, [3, 6–14] were conducted using cross-sectional statistical methods which generally have limited capacity to discover new and unanticipated patterns that are hidden in data and identify cause and effect relationships [6, 10, 15]. These studies did not also include features that lead to anemia such as the history of birth, history of abortion, history of the place of delivery, history of malaria, and nutritional variables. i.e. the factors that contribute to the occurrence of anemia among pregnant women weren’t thoroughly studied. In such situations, new technologies like machine learning algorithms may help to discover hidden patterns [16]. There were machine learning-related works such as [17–20]. However, these studies aimed at developing a predictive model, but did not identify the most determinant risk factors, and generate rules that allow the development of evidence-based strategies and policies toward preventing and/or reducing anemia among pregnant women in Ethiopia. This study, hence, aims to develop a model that predicts the level of anemia among pregnant women using homogeneous ensemble machine learning algorithms by investigating the following research questions: (1) what is the underlying structure of anemia among pregnant women in Ethiopia? (2) Which homogeneous ensemble of machine learning algorithms is suitable for predicting the level of anemia among pregnant women in Ethiopia? (3) What are the associated risk factors that influence the occurrence of anemia among pregnant women in Ethiopia? (4) What are the important rules that may shape strategies and policies towards preventing and/or reducing anemia among pregnant women in Ethiopia?

The rest of this document is organized as follows: Section II presents related works, Section III discusses materials and methods used, Section IV mentions experimental setup and result discussion, and Section V presents the conclusion.

## Related works

Several studies such as [3, 6–14] investigated the status of anemia among pregnant women and its determinant factors in different parts of Ethiopia using cross-sectional statistical methods. They used bivariate and multivariate logistic regression methods. However, cross-sectional statistical methods usually have limited capacity to discover new and unanticipated patterns and identify cause and effect relationships that are hidden in data [6, 10, 15]. Most of these previous studies used local clinical data that covered limited geographical areas like a single city or town only, employed small data set less than 500 instances/records, and focused on one of the risk factors like socioeconomic, demographic, nutritional, and reproductive, apart from health-related variables. Some of them also identified the determinant risk factors of anemia among pregnant women who followed first antenatal care. These studies did not include features, such as history of birth, history of abortion, history of place of delivery, history of malaria, and nutritional variables. I.e. the factors that contribute to the occurrence of anemia among pregnant women weren’t thoroughly studied. Dithy and Krishnapriya [17] predicted anemia among pregnant women using ANN and gausnominal classification algorithm with an accuracy of 0.65% and 0.74%, respectively. Dithy and Krishnapriya [18] tried to classify anemia in pregnant women using random prediction (Rp) classification algorithm and achieved an accuracy of 0.65%, 0.76%, 0.826%, and 0.92% with ANN, gausnominal, vector neighbor, and random, respectively. Nevertheless, these studies did not consider all potential features that are discussed in section I, which helps to take holistic interventions. Furthermore, [17–20] aimed to construct a predictive model, but they did not identify risk factors, and extract rules which are important to make evidence-based strategies, policies and interventions towards preventing and/or reducing anemia among pregnant women in Ethiopia. This study, hence, motivated to fill these gaps by constructing a predictive model, identifying risk factors, extracting relevant rules, designing an innovative artifact and deploying the predictive model for potential users.

## Materials and methods

### Data collection

The data used in this research was extracted from the Ethiopian Demographic Health Survey (EDHS) which was collected by the Ethiopian central statistical agency in 2005, 2011, and 2016, in the five-year interval.

### Data preprocessing

The extracted datasets consist of a total of 11,174 instances with 34 features. As all these features are not relevant for developing a predictive model that can predict the level of anemia among pregnant women in the case of Ethiopia, data preprocessing techniques such as data cleaning, data transformation, handling class imbalance, removal of quasi-constant features, and feature selection methods were applied. The missing values were handled using mode imputation techniques for categorical data. Redundant data were removed manually. The quasi-constant features were not directly removed, but we have constructed one feature and combined them into one. There were features which have several distinct values and need to be transformed for mining purposes; such as features with more categorical values such as the source of drinking water, body mass index, wealth index, marital status, and household members were transformed into discrete values using binning discretization mechanisms. Then, feature selection methods were applied to select the relevant features which are important for the further process [21]. In this study, two types of feature selection methods (filter, and wrapper) were employed to see which one produces better results. As a result, the step-forward feature selection method performs better than others, see Table 1 which shows the list of features ordered based on their importance in predicting anemia among pregnant women. Besides, domain experts (antenatal care professionals from the University of Gondar specialized hospital) recommended additional seven features, see Table 2. After conducting all the required data preprocessing tasks, a total of 29,104 instances with 23 features were considered for further analysis and prediction model development. Finally, the dataset was divided into training and testing datasets following an 80/20% ratio. The class level of the training dataset was imbalanced which was treated using the synthetic minority over-sampling technique (SMOTE) to avoid loss of valuable information [22, 23].

**Table 1:** Feature selection results

	Mutual information feature selection	Chi^2^ feature selection	F class if feature selection	Step forward feature selection	Step backward feature selection
0	Age in 5-year groups	Region	Region	Age in 5-year groups	Age in 5-year groups
1	Region	Highest educational level	Type of place of residence	Region	Region
2	Number of antenatal care visits	Source of drinking water	Highest educational level	Number of antenatal care visits	Number of antenatal care visits
3	Highest educational level	Religion	Source of drinking water	Source of drinking water	Highest educational level
4	Religion	Frequency of reading newspaper or magazine	Religion	Religion	Source of drinking water
5	Frequency of watching television	Frequency of listening to radio	Frequency of watching television	Number of household members	Religion
6	Duration of current pregnancy	Frequency of watching television	Duration of current pregnancy	Frequency of listening to radio	Number of household members
7	Birth history	Currently breastfeeding	Current pregnancy wanted	Duration of current pregnancy	Frequency of listening to radio
8	History of contraceptive use	Mosquito bed net	History of contraceptive use	birth history	Duration of current pregnancy
9	Body mass index	Husband/partner's education level	Husband/partner's education level	Current pregnancy wanted	birth history
10	Husband/partner's education level	Respondent's occupation	Respondent's occupation	History of contraceptive use	Current pregnancy wanted
11	Husband/partner's occupation	History of the place of delivery	History of the place of delivery	Body mass index	Body mass index
12	Respondent's occupation	Iron tablet during pregnancy	Iron tablet during pregnancy	Husband/partner's education level	Husband/partner's education level
13	History of the place of delivery	Had diarrhea recently	Had diarrhea recently	Husband/partner's occupation	Husband/partner's occupation
14	Vitamin a in last 6 months	Vitamin a in last 6 months	Vitamin a in last 6 months	Respondent's occupation	Respondent's occupation
15	Wealth index combined	Wealth index combined	Wealth index combined	Wealth index combined	Wealth index combined
Accuracy with RF	89.091221	76.120941	82.85518	0.91813755	0.917751321

**Table 2:** Features selected by domain experts

No	Features	Feature descriptions
1	m49a	Take drug for malaria during pregnancy
2	H34	Take Vitamin A
3	V106	Highest educational level
4	M15	History of Place of delivery
5	m45	Iron tablet during pregnancy
6	V228	History of terminating a pregnancy
7	V404	Breastfeeding status

The SMOTE method employs a KNN technique, choosing K-nearest neighbors and joining them to produce synthetic samples in space [22, 23]. The algorithm calculates the distance between the feature vectors and their closest neighbors and didn’t generate duplicates, but rather creating synthetic data points that are slightly different from the original data points [22, 23]. The algorithm takes the feature vectors and its nearest neighbors, computes the distance between these vectors. Due to this, we have used SMOTE for handling the imbalances of the dataset.

#### Predictive model development

To construct a model that predicts the level of anemia among pregnant women in the case of Ethiopia, homogeneous ensemble machine learning algorithms such as extreme gradient boosting, random forest, and cat boost algorithms without applying class decomposition and with applying one versus one and one versus rest class decomposition were selected for an experiment. To show that homogeneous ensemble algorithms can perform better than other supervised machine learning algorithms, another model was developed using decision tree algorithms. For developing the predictive models, 23 features selected by the step forward feature selection method and three domain experts (1 MSc and 2 BSc holders) who are working at the University of Gondar Referral Hospital as antenatal care professionals were used. Grid search was implemented to tune the hyperparameters of each algorithm, as the performance of the algorithm highly depends on the selection of hyperparameter, which has always been a crucial step in the process of machine learning model development [24–26]. The performance of each predictive model was evaluated using accuracy, precision, recall, F1- score, K-fold cross-validation, and ROCAUC.

**Fig. 1:**
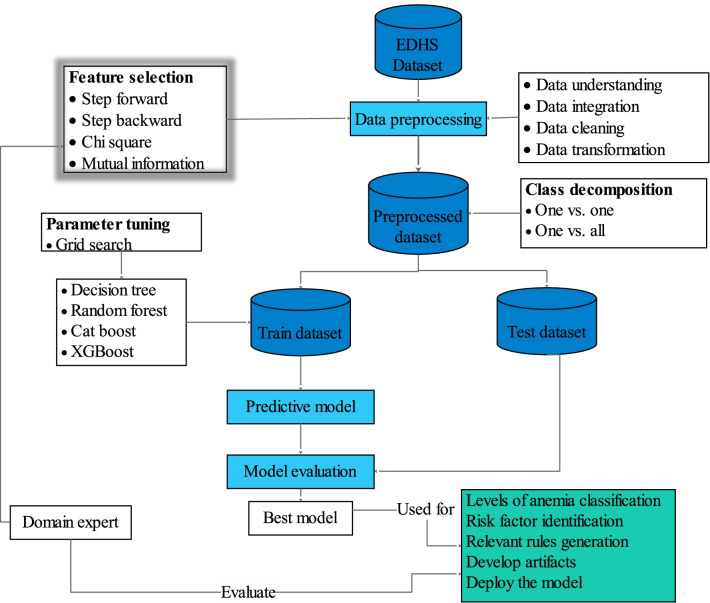
Proposed model development workflow/architecture

Figure 1 presents the work flows and methods that were followed in this study to develop a predictive model, select the best-performed model, identify risk factors, generate relevant rules, design artifacts, and deploy the final model for potential users.

## Experimental setup and results discussion

Here below results are discussed based on the research questions.

### What is the underlying structure of anemia among pregnant women in Ethiopia?

**Fig. 2:**
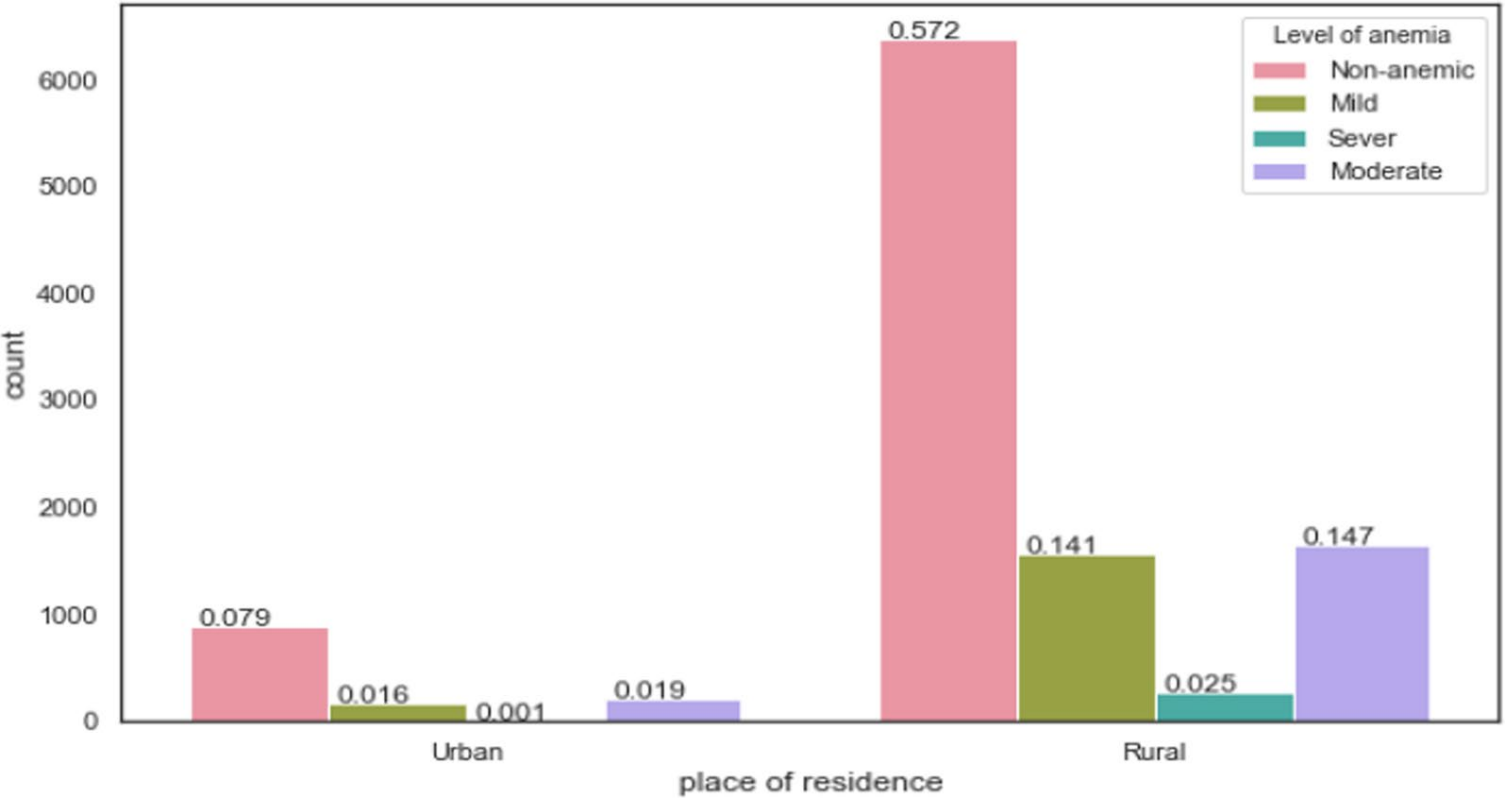
Prevalence of Anemia based on place of residence

To show the underlying structure of anemia among pregnant in the case of Ethiopia, a descriptive statistical technique was used by considering the age, place of residence, region, antenatal care visit, history of the place of delivery, history of terminating the pregnancy, and wealth index with the anemia level. As a result, pregnant women who live in the rural areas of Ethiopia are highly affected by anemia, and in the rural areas of Ethiopia the level of non-anemic, mild, severe, and moderate anemia is 57.2%, 14.1%, 2.5%, and 14.7%, respectively, see Fig. 2 below. This shows that every level of anemia in rural areas of Ethiopia was higher than in the urban area of Ethiopia.

**Fig. 3:**
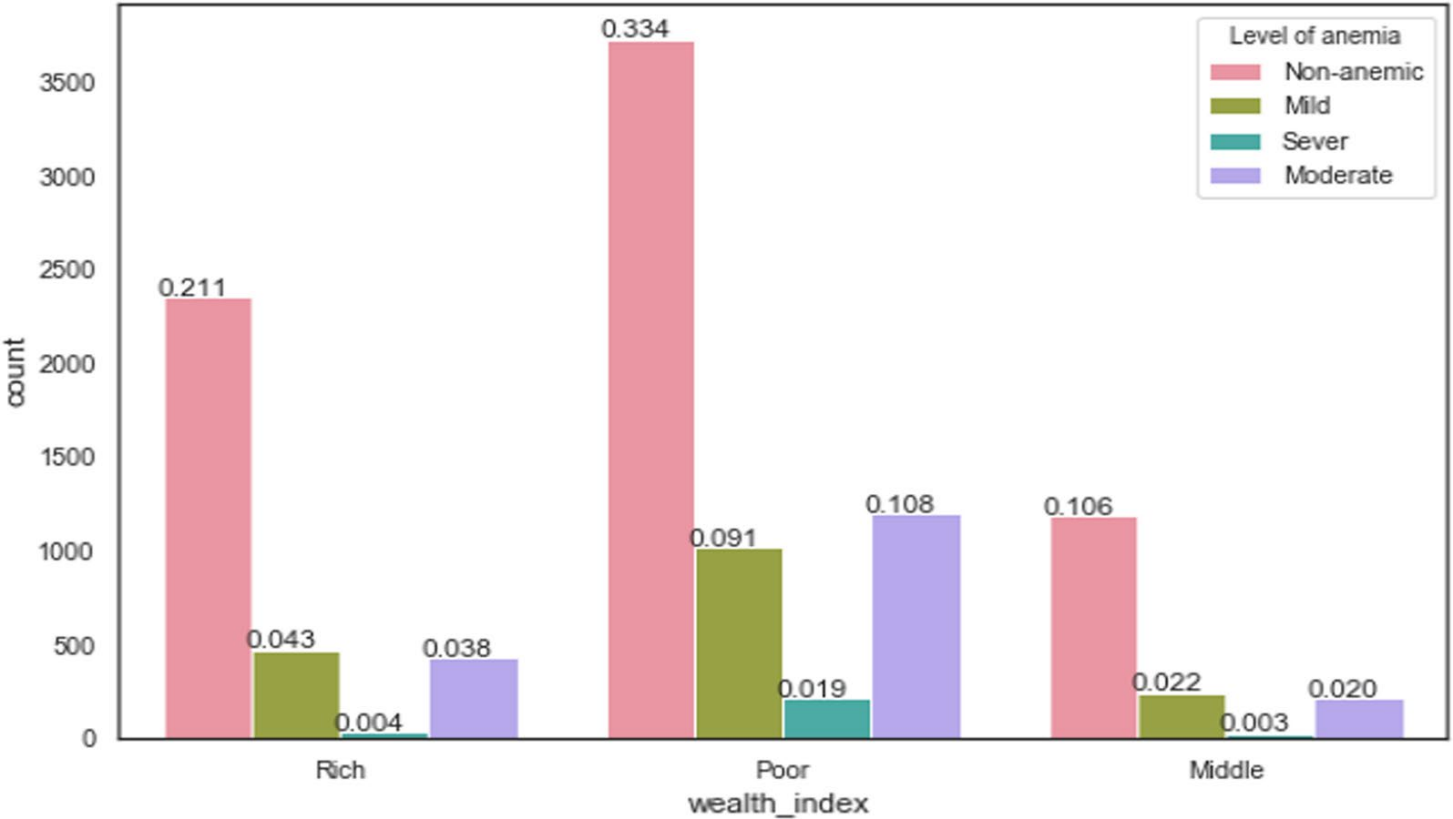
Prevalence of anemia based on wealth index status

Figure 3 illustrate that pregnant women with poor economic status were highly affected by anemia and pregnant women with poor wealth index status were higher than other wealth index status in every level of anemia.

**Fig. 4:**
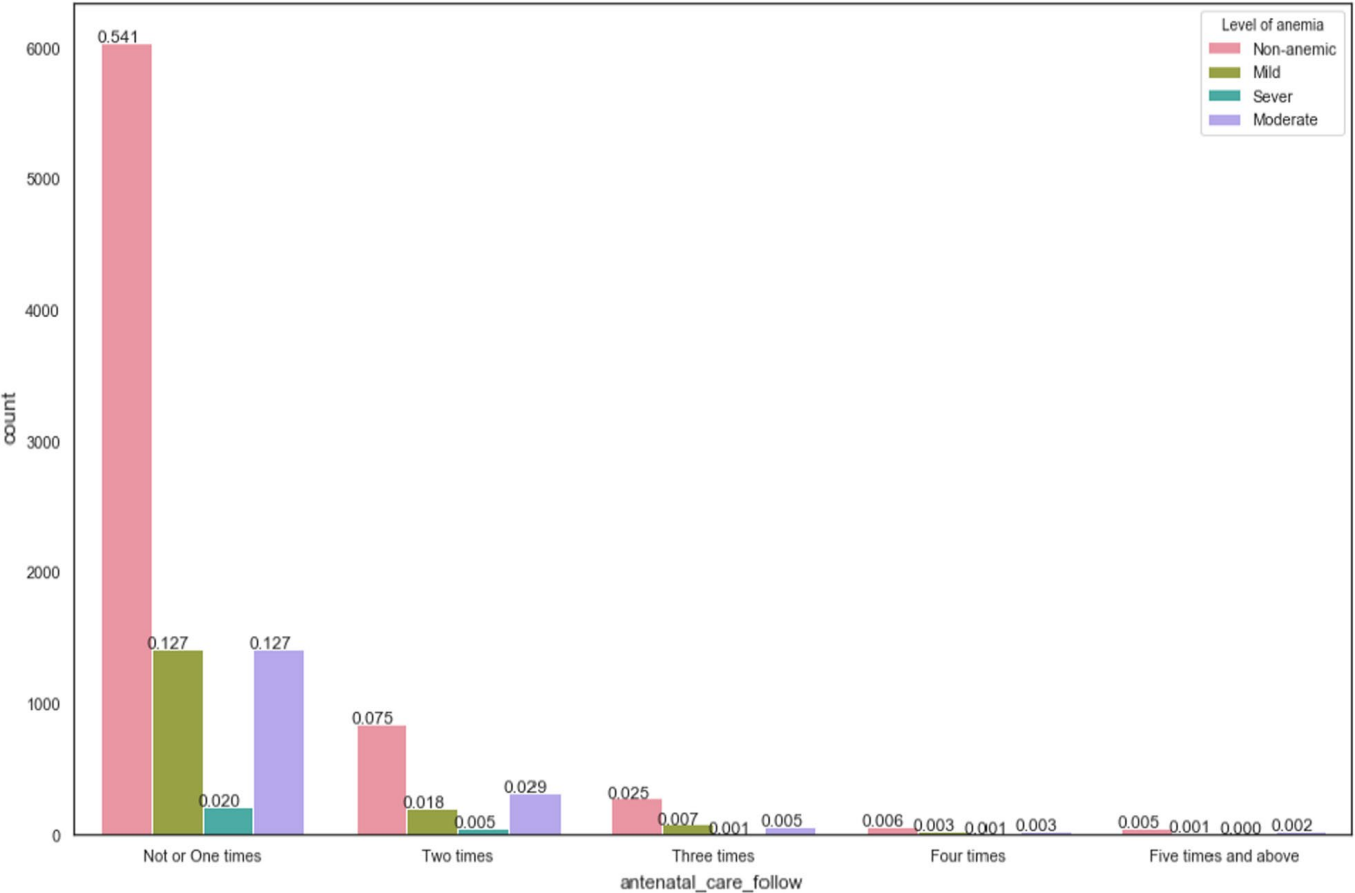
prevalence of anemia among pregnant women based on antenatal care follow-up

Pregnant women who didn’t follow or follow antenatal care one time only during pregnancy were highly affected by anemia. Whereas, pregnant women who follows antenatal care often has low probability of having anemia, see Fig. 4 here below.

**Fig. 5:**
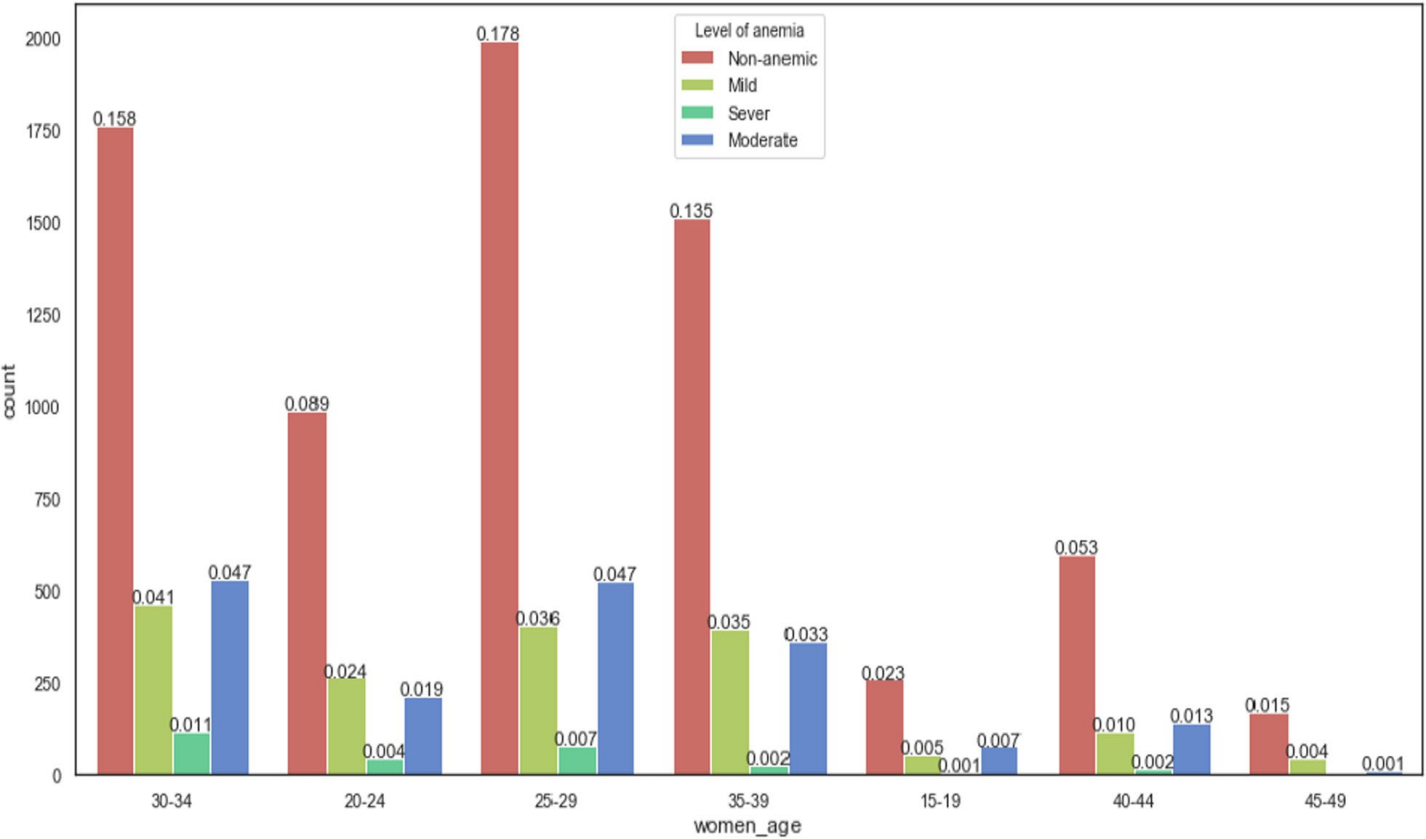
prevalence of anemia among pregnant women based on pregnant women's age group

**Fig. 6:**
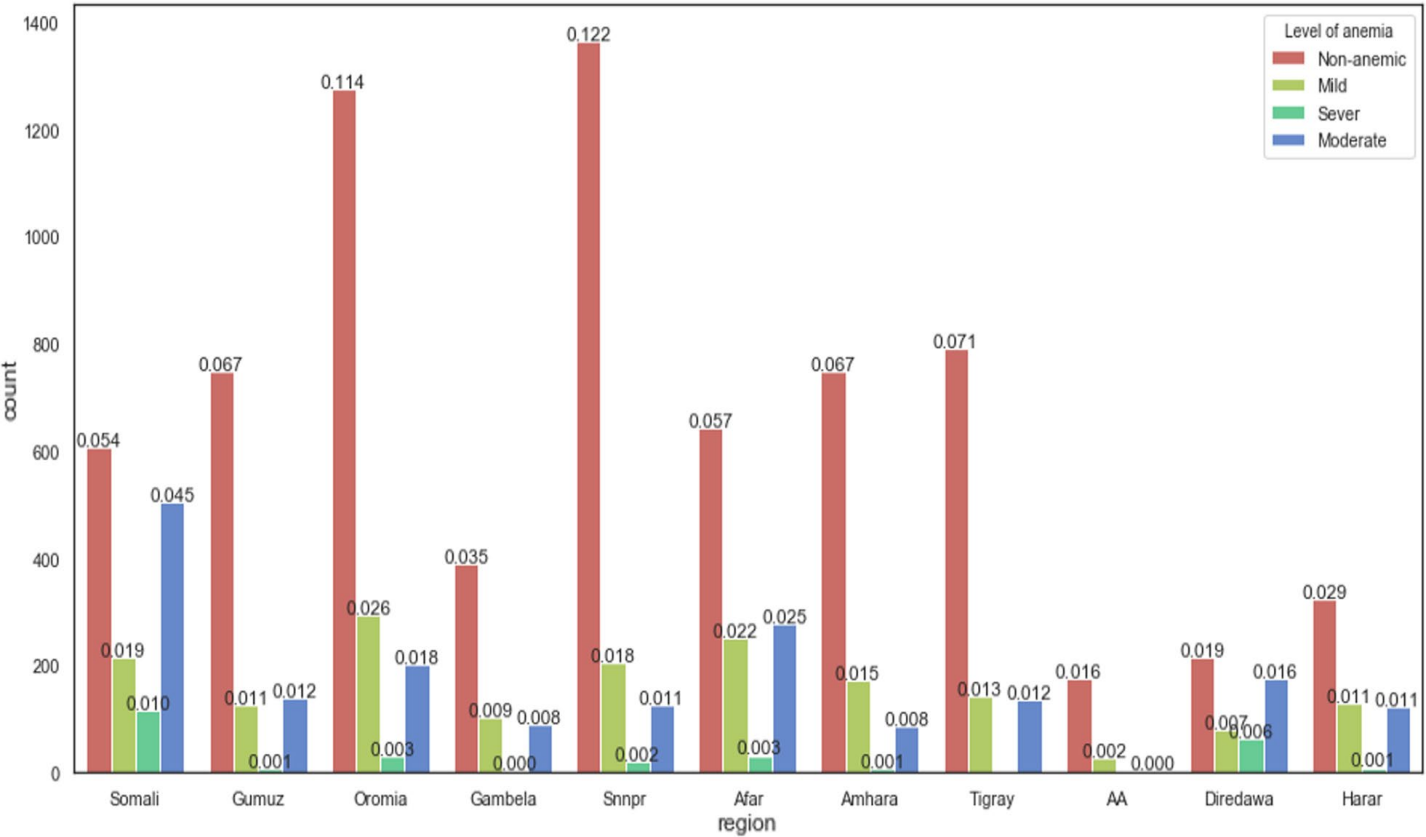
prevalence of anemia among pregnant women based on region

Figure 5 dipicts the anemia level distribution among pregnant women in different age groups and, made evident that the pregnant women between the ages of 30–34 were severely affected by anemia. Besides, the Ethiopian regions, such as Somalia, Afar, Dire Dawa and Southern Nations, Nationalities, and People's Region (SNNPR) were highly affected by anemia, see Fig. 6.

### Which homogeneous ensemble of machine learning algorithms is suitable for predicting the level of anemia among pregnant women in Ethiopia?

To answer this question, twelve experiments using three homogeneous ensemble machine learning algorithms namely random forest, extreme gradient boosting, and cat boost with class decomposition (by using one versus one and one versus the rest), and without class decomposition were conducted. To show that homogeneous ensemble algorithms can perform better than other supervised machine learning algorithms, we have also conducted an experiment using decision tree algorithms. The experiments showed that the model that was developed using the cat boost algorithm with one versus the rest class decomposition performs better in predicting the level of anemia among pregnant women in the case of Ethiopia with 97.6% of accuracy, 97.59% of precision, 97.57% of recall, 97.58% of f1_score, and, 99.9% of roc see Table 3 below, with parameters of (depth = 10, iterations = 300, l2_leaf_reg = 1, learning_rate = 0.15) which were tuned using grid search. A random forest algorithm with parameters (criterion = 'entropy', max_features = 'sqrt', min_samples_split = 3, n_estimators = 500, random_state = 0, max_depth = 20, max_leaf_nodes = 400, n_jobs = −1) performs less than cat boost algorithms, extreme gradient boosting algorithms with  default parameters, and decision tree algorithm with parameters (criterion = 'entropy',max_features = 'sqrt', min_samples_split = 12, random_state = 0, max_depth = 30, max_leaf_nodes = 600) performs less performance than all other algorithms, see Table 3 for detailed results.

**Table 3:** Model performance

ML algorithm	Parameters	Evaluation metrics	Without class decompositions (%)	With one vs. one class decomposition (%)	With one vs. rest class decomposition (%)
Decision tree	criterion = 'entropy',max_features = 'sqrt',min_samples_split = 12,random_state = 0,max_depth = 30, max_leaf_nodes = 600	Accuracy	79.38	89.88	89.09
		precision	79.09	89.81	89.01
		Recall	79.21	89.77	88.98
		F1_score	79.03	89.71	88.96
		Cross-validation	68.48	84.27	83.17
		ROC	95.6	95.6	95.6
Random forest	criterion = 'entropy', max_features = 'sqrt', min_samples_split = 3, n_estimators = 500, random_state = 0, max_depth = 20, max_leaf_nodes = 400, n_jobs = -1	Accuracy	91.34	94.4	94.4
		Precision	91.32	94.36	94.37
		Recall	91.28	94.35	94.35
		F1_score	91.25	94.34	94.34
		Cross-validation	81.23	89.37	88.18
		ROC	99	99	99.43
Cat boost	depth = 10, iterations = 300, l2_leaf_reg = 1, learning_rate = 0.15	Accuracy	97.08	97.44	**97.595**
		Precision	97.09	97.438	**97.596**
		Recall	97.05	97.418	**97.574**
		F1_score	97.06	97.422	**97.58**
		Cross-validation	95.94	96.478	**96.482**
		ROC	99.9	99.94	**99.9**
Extreme gradient Boost	max_depth = 3, learning_rate = 0.1, n_estimators = 100, silent = True, objective = 'binary: logistic’ booster = 'gbtree', n_jobs = 1, nthread = None	Accuracy	94.26	95.21	94.54
		Precision	94.27	95.20	94.53
		Recall	94.20	95.16	94.48
		F1_score	94.20	95.16	94.48
		Cross-validation	88.86	91.73	89.72
		ROC	99.53	99.53	99.54

### What are the associated risk factors that influence the occurrence of anemia among pregnant women in the case of Ethiopia?

To answer this question, feature importance analysis was performed using the model that was developed with the best performing algorithm which is cat boost. As a result, we have identified that duration of current pregnancy, age in 5-years group, source of drinking water, history of contraceptive use, accupation, number of household members, weath index, frequency of listing to the radio, partner’s education level, region, partner’s education accupation, and birth history are highly associated with the level of anemia among pregnant women in Ethiopia. Table 4 shows the most important risk factors that determines the level of anemia among pregnant women in Ethiopia.

**Table 4:** Identified risk factors with best fit model and feature importance

Feature	Values	Feature	Values
Duration of current pregnancy	10.3953193	Current pregnancy wanted	3.838873474
Age in 5-year groups	9.69394377	Body mass index	2.787116569
Source of drinking water	8.99369175	Number of ANC visits	2.600944933
History of contraceptive use	6.61405164	Highest educational level	2.419310637
Pregnant woman’s occupation	6.12946203	History of terminating a pregnancy	0.849814164
Number of household members	5.85914199	Currently breastfeeding	0.732357678
Wealth index	5.63211101	Type of place of residence	0.576997215
Frequency of listening to the radio	5.16045505	Vitamin A in last 6 months	0.356953114
Husband/partner's education level	5.02943094	During pregnancy, given or bought iron tablets/syrup	0.046775106
Region	4.3314029	History of Place of delivery	0.010932682
Husband/partner's occupation	3.96855455	During pregnancy took: sp/ fansidar for malaria	0.00058328
Birth history	3.87177534		

### What are the important rules that can be generated from the predictive model?

To answer this question, we used all the features that were used to develop the predictive model and generate important rules by using the best-performed model (cat boost algorithms with one versus rest class decompositions) for the level of anemia among pregnant women in the case of Ethiopia. Then these rules, which are presented here below, were also validated by three midwiferies (1 MSc and 2 BSc holders) who are working at the University of Gondar Referral Hospital. We believe that these rules are vital to develop strategy and policy toward preventing and/or controlling anemia among pregnant women in Ethiopia.

**RULE1,** IF given iron tablet or syrup during pregnancy =  = 'No' ^vitamin A in last 6 months =  = 'No' ^ during pregnancy took sp fansidar for malaria =  = 'No' ^region =  = 'Somali' ^currently breastfeeding =  = 'No' AND ^place of residence =  = 'rural' ^Duration of current pregnancy =  = 'seven-nine-week' ^ current pregnancy wanted =  = 'Yes' ^ respondents occupation =  = 'did not work' ^ history of place of delivery =  = 'Home' ^ age =  = 'thirty—thirty four' ^ educational level =  = 'no education' ^ husband educational level =  = 'no education' ^ number of household =  = 'six-ten' ^ history of terminating pregnancy =  = 'No' ^ body mass index =  = 'normal' ^ husband occupation =  = 'did not work' THEN anemia level =  = 'sever'.

**RULE2,** IF given iron tablet or syrup during pregnancy =  = 'No' ^ vitamin A in last 6 months =  = 'No' ^ during pregnancy took sp fansidar for malaria =  = 'No' ^ region =  = 'Somali' ^ currently breastfeeding =  = 'No' ^ place of residence =  = 'rural' ^ Duration of current pregnancy =  = 'seven-nine-week' ^ current pregnancy wanted =  = 'Yes' ^ respondents occupation =  = 'did not work' ^ place of delivery =  = 'Home' ^ age =  = 'thirty—thirty four' ^ educational level =  = 'no education' ^ husband educational level =  = 'no education' ^ number of household =  = 'six-ten' ^ History of terminating pregnancy =  = 'No' ^ body mass index =  = 'normal' ^ husband occupation =  = ' agricultural—employee' ^ source of water =  = 'pure' ^ history of contraceptive use =  = 'Yes' THEN anemia level =  = 'none anemic'.

**RULE3,** IF given iron tablet or syrup during pregnancy =  = 'No' ^ vitamin A in last 6 months =  = 'No' ^ during pregnancy took sp fansidar for malaria =  = 'No' ^ region =  = 'Somali' ^ currently breastfeeding =  = 'No' ^ place of residence =  = 'rural' ^ Duration of current pregnancy =  = 'seven-nine-week' ^ current pregnancy wanted =  = 'Yes' ^ respondents occupation =  = 'did not work' ^ history of place of delivery =  = 'Home' ^ age =  = 'thirty—thirty four' ^ educational level =  = 'no education' ^ husband educational level =  = 'no education' ^ number of household =  = 'six-ten' ^ history of terminating pregnancy =  = 'No' ^ body mass index =  = 'normal' ^ husband occupation =  = ' agricultural—employee' ^ source of water =  = 'not pure' ^ history of contraceptive use =  = 'Yes' THEN anemia level =  = 'Moderate’.

Finally, the predictive model was deployed on the cloud for potential users. The artifact was designed using a Python module called Flask with HTML and deployed on Heroku. All potential users can access the predictive model via https://anemia-level-prediction-model.herokuapp.com/ to predict a pregnant woman’s level of anemia.

## Conclusion

Anemia is a global public health issue that affects a wide range of people of all ages. Anemia during pregnancy is one of the risk factors for poor pregnancy outcomes, such as low birth weight, preterm birth, prematurity stillbirth, intrauterine growth restriction, and impaired cognitive development. This study aimed to develop a predictive model for the level of anemia among pregnant women in the case of Ethiopia by using homogeneous ensemble machine learning algorithms. This study was conducted using design science methodology. The proposed model was constructed using homogeneous ensemble machine learning algorithms namely random forest, extreme gradient boosting, and cat boost algorithms with class decomposition methods and without class decomposition methods. To conduct this study, we have done a total of twelve experiments. The cat boost algorithm with one versus all class decomposition has registered the highest performance with 97.6% of accuracy, 97.59% of precision, 97.57% of recall, 97.58% of f1_score, and 96.48% of cross-validation. We have identified the determinant risk factors by conducting a feature importance analysis on the best-performed algorithms. Some of the most determinant risk factors were duration of current pregnancy, age in five years group, source of drinking water, history of contraceptive use, respondent’s occupation, and several household members. The most important rules were also generated using the best fit model for developing policies and interventions toward maintaining anemia among pregnant women.

Finally, we recommend that future researchers conduct a predictive model for pregnant women that predicts which type (Vitamin deficiency anemia, Anemia of inflammation, Aplastic anemia, or iron-deficiency anemia) of anemia has occurred within the pregnant women. A predictive model that can predict the level of anemia among neonatal based on maternal determinants during pregnancy and the determinant risk factors anemia over time.

## Data Availability

The datasets generated and/or analysed during the current study are available in the ‘Anemia level’ repository, https://github.com/belzman/Anemia_level.

## Abbreviations

ANN: Artificial Neural Network
EDHS: Ethiopian Demographic and Health Survey
Hb: Hemoglobin
HTML: Hypertext Markup Language
LBW: Low Birth Weight
RP: Random Prediction
SMOTE: Synthetic Minority Over-sampling Technique
SNNPR: Southern nation and nationality of people
